# PIF* promotes brain re-myelination locally while regulating systemic inflammation- clinically relevant multiple sclerosis *M.smegmatis* model

**DOI:** 10.18632/oncotarget.15662

**Published:** 2017-02-24

**Authors:** Giuseppe Migliara, Martin Mueller, Alessia Piermattei, Chaya Brodie, Michael J. Paidas, Eytan R. Barnea, Francesco Ria

**Affiliations:** ^1^ Università Cattolica del S. Cuore, Institute of General Pathology, Largo Francesco Vito, 100168 Rome, Italy; ^2^ Department of Obstetrics and Gynecology, University of Bern, 3010, Bern, Switzerland; ^3^ Department of Obstetrics, Gynecology and Reproductive Sciences, Yale Women and Children's Center for Blood Disorders and Preeclampsia Advancement, Yale University School of Medicine, FMB 3398, New Haven, CT 06520-8063, USA; ^4^ Department of Neurosurgery, Henry Ford Hospital, Detroit, MI 48202, USA; ^5^ Society for the Investigation of Early Pregnancy (SIEP), Cherry Hill, NJ 08003, USA; ^6^ BioIncept, Cherry Hill, NJ 08003, USA; ^7^ Present address: Department of Public Health and Infectious Diseases, Sapienza University of Rome, 00185 Rome, Italy

**Keywords:** preImplantation factor, neuroprotection, neuroregeneration, M. smegmatis bacteria, RR-EAE clinically-relevant model

## Abstract

Neurologic disease diagnosis and treatment is challenging. Multiple Sclerosis (MS) is a demyelinating autoimmune disease with few clinical forms and uncertain etiology. Current studies suggest that it is likely caused by infection(s) triggering a systemic immune response resulting in antigen/non-antigen-related autoimmune response in central nervous system (CNS). New therapeutic approaches are needed. Secreted by viable embryos, PreImplantation Factor (PIF) possesses a local and systemic immunity regulatory role. Synthetic PIF (PIF) duplicates endogenous peptide's protective effect in pre-clinical autoimmune and transplantation models. PIF protects against brain hypoxia-ischemia by directly targeting microglia and neurons. In chronic experimental autoimmune encephalitis (EAE) model PIF reverses paralysis while promoting neural repair. Herein we report that PIF directly promotes brain re-myelination and reverses paralysis in relapsing remitting EAE MS model. PIF crosses the blood-brain barrier targeting microglia. Systemically, PIF decreases pro-inflammatory IL23/IL17 cytokines, while preserving CNS-specific T-cell repertoire. Global brain gene analysis revealed that PIF regulates critical Na^+^/K^+^/Ca^++^ ions, amino acid and glucose transport genes expression. Further, PIF modulates oxidative stress, DNA methylation, cell cycle regulation, and protein ubiquitination while regulating multiple genes. In cultured astrocytes, PIF promotes BDNF-myelin synthesis promoter and SLC2A1 (glucose transport) while reducing deleterious E2F5, and HSP90ab1 (oxidative stress) genes expression. In cultured microglia, PIF increases anti-inflammatory IL10 while reducing pro-inflammatory IFNγ expression. Collectively, PIF promotes brain re-myelination and neuroprotection in relapsing remitting EAE MS model. Coupled with ongoing, Fast-Track FDA approved clinical trial, NCT#02239562 (immune disorder), current data supports PIF's translation for neurodegenerative disorders therapy.

## INTRODUCTION

Immune balance is a key aspect of the physiological development and function of an organism. Imbalance of this essential homeostasis can lead to autoimmune disease (AD). Similarly, effective embryo-maternal crosstalk leading to controlled immunomodulation is essential for successful outcome of pregnancy [1]. The semi-allogeneic fetus (transplant) is not rejected by the mother, achieving “transplant tolerance”. Concurrently, protection against pathogens (regulated immunity) remains largely preserved thus reflecting a close to optimal immune milieu. Seemingly, paradoxically, in the case of an immune challenge, such as AD, the mechanisms of this maternal immune status are able to mitigate or even reverse the AD. Several ADs, such as rheumatoid arthritis [2] and multiple sclerosis (MS) [3, 4], actually improve during pregnancy, while miscarriage may exacerbate their symptoms [3]. Protection against AD, however, is pregnancy-specific and only temporary, since shortly following parturition they tend to flare up [4–8]. Not surprisingly, the search for pregnancy-specific compounds, that are suitable for clinical use, is ongoing [4, 5].

Ideally, such a compound would regulate both local and systemic immunity, in a safe manner, since AD impairs both. Specifically, for MS/neuroinflammation, such a drug would promote brain myelination and reverse paralysis through integrated local and systemic action. Such is the premise of the use of PreImplantation Factor (PIF*). PIF is an endogenous peptide, secreted by viable embryos and absent in non-viable ones. PIF is expressed from the earliest stages of gestation and is essential to pregnancy [9–13]. Further, PIF protects and supports embryo development [14] and promotes implantation and trophoblast invasion [12, 15–18]. Mechanistically, PIF regulates systemic immunity, targeting the innate arm, while only minimally affecting quiescent lymphocyte activity. Following peripheral blood mononuclear cell activation, PIF blocks the mixed lymphocyte reaction and proliferation, leading to a Th2 cytokine bias typical of pregnancy while not concurrently suppressing the beneficial Th1 type response [17, 19, 20]. In addition, PIF reduces K+ flux by targeting the cortisone binding site (Kv1.3b channel) [21] while targeting PDI/thioredoxin and heat shock proteins to prevent oxidative stress and protein misfolding [19]. PIF regulates systemic immunity [19, 20, 22]. PIF reduces the cytotoxicity of NK cells as well [9, 17, 19].

Consistent with the view that a pregnancy derived compound PIF can also be effective beyond pregnancy, a synthetic PIF (PIF) was successfully used in non-pregnant animal models. For example, PIF administration prevents development of Type 1 diabetes mellitus [23], atherosclerosis [21], and graft vs. host disease [24] as well protects against acute radiation induced pathologies [25]. In neurodegeneration models, PIF passes the blood-brain barrier, protects the immature brain after injury, and targets both the microglia and neurons [26, 27]. In an adult MS-experimental autoimmune encephalomyelitis (EAE) model, PIF reverses severe paralysis while promoting/maintaining neural repair long-term [28]. All in all, PIF provides neuroprotection from the embryonal stage (against adverse maternal environment), to the newborn and adult through an integrated local and systemic effect [1, 18].

Current MS therapy aims to reduce the progressive neural damage and minimize its clinical manifestations. For acute attacks, steroids and β-interferon are used [29]. To reduce the frequency of paralytic attacks glatiramer acetate, natalizumab, linomide, fingolimod, and dimethyl fumarate are useful [30–34]. For the treatment of progressive MS, mitoxantrone and cladribine may be used [35]. Stem cell transplantation for MS therapy is still experimental. The customarily used preclinical models that led to the approved drugs do not fully represent the MS spectrum. EAE, for example, can be induced by various CNS peptides or by transfer of encephalitogenic T-cells [36–38], each only reproduces a specific aspect of clinical MS. Thus, no single current EAE model accurately recapitulates the clinical MS spectrum [39]. In MS, genetics also plays a significant role as seen by the association between MHC region immune responses and the disease [40, 41]. However, the low concordance between mono-zygotic twins indicates a predominantly important role also for environmental factors. One crucial factor of these is bacterial infection. For a classical EAE, mycobacterium-derived antigen motifs are required leading to polarization of CD4+ and CD8+ T-cells, and trafficking of CNS specific T cells [42–44]. Notably, MS improvement (in EAE models) was noted both in the white and grey matter of the brain [45, 46]. To test PIF induced protective effects, we used a chimeric M. smegmatis bacterium producing proteolipid protein peptide (PLP) 139-151 (p139) fused with MPT64 to induce a clinically-relevant RR-EAE model [43]. We examined PIF effects on brain myelination, clinical score and global genome expression. Further, PIF effects on systemic cytokine expression and lymph node T-cell repertoire was determined. From a clinical point of view, cGMP PIF (human grade) is already being used clinically in FAST-TRACK awarded, FDA-approved, University-sponsored clinical trials to assess PIF drug safety and efficacy in an autoimmune disorder (ClinicalTrials.gov NCT02239562).

## RESULTS

### Both continuous and intermittent PIF administration consistently ameliorate RR-EAE

**Figure 1:**
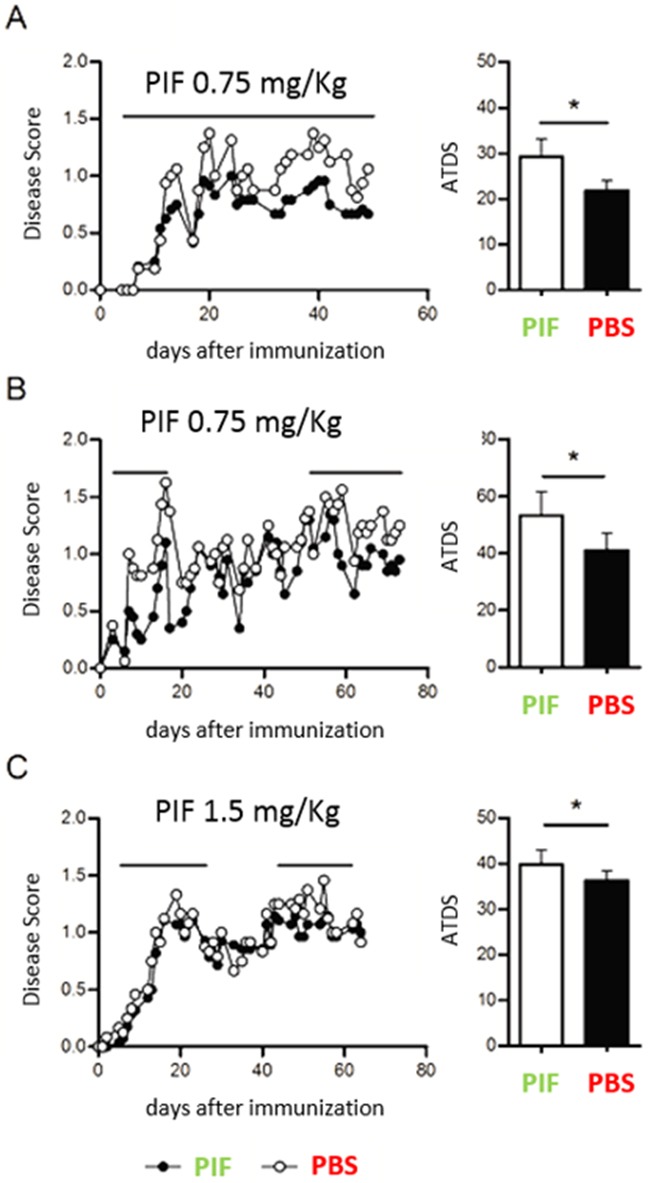
Continuous and intermittent PIF administration reduces clinical score (RR-EAE model) SJL mice (4-7 per group) were infected sc with 4×106 CFU of live recombinant M Smegmatis expressing a recombinant chimeric protein MPT64-PLP139-151 (rMSp139), as previously published [43]. Starting on day 3 after infection, mice were treated daily with PIF (closed symbols and black bars) or vehicle only (PBS, open symbols and white bars). **(A)** Disease course and average total score of disease in mice treated continuously until day 50 after infection with 0.75 mg/Kg of PIF (n=6) or with vehicle only (n=4) **(B)** Disease course and average total score of disease in mice treated from day 3 until day 18 and then from day 51 until day 70 with 0.75 mg/Kg of PIF (n=6) or with vehicle only (n=4). **(C)** Disease course and average total score of disease in mice treated from day 3 until day 25 and then from day 51 until day 65 with 1.5 mg/Kg of PIF (n=7) or with vehicle only (n=6). Disease score was monitored by two independent examiners, blinded with respect to treatment. *p< 0.05; Mann-Whitney test. PIF: PreImplantation Factor, ATDS: average total disease score.

Given that early MS can start as an acute paralytic attack and that PIF was shown to prevent and reverse paralysis in a classic antigen driven EAE model [28], we tested *continuous* PIF administration using the infectious/inflammatory RR-EAE model (Figure 1A). The RR-EAE model is utilitarian for anti-MS drug development and although M. smegmatis bacteria are rapidly cleared, neuroinflammation becomes progressive, reflecting advanced stage disease [43]. In addition, we also have shown that the induction of EAE is dependent on the ability of the infection to drive a CNS-specific immune response, since infection with a recombinant M. smegmatis not expressing Plp 139-151 does not result in clinical EAE. To mimic a clinical approach, we treated mice with PIF (0.75 mg/kg i.p daily) or PBS [28]. As expected, starting on the third day post-inoculation PIF significantly decreased the resulting clinical score (Figure 1A) of injured animals. Maximal decrease was present at the acute (early) disease phase. Mean clinical score and average total disease score (ATDS) were significantly lower compared to control group. The maximal decrease was noted after a couple of days of PIF administration, at the acute phase of disease. The mean clinical score was lower than in vehicle treated controls throughout and until the end of the experiment. The ATDS was significantly decreased by PIF administration (PBS 29.25 vs PIF 21.75; p= 0.01, Wilcoxon-Mann-Whitney test). The composite effect of PIF on AUC are shown (Supplementary Figure 1). Together, continuous PIF administration can consistently ameliorate post-infective acute paralysis.

Again, attempting to mimic human MS, which has a relapsing-remitting course, we next examined whether intermittent PIF administration, as needed when symptomatic, is also effective (Figure 1B). After RR-EAE induction PIF treatment was only during the active state of disease while interrupting treatment during the remission period. Again, PIF significantly decreased the clinical score compared with controls (ATDS: PBS 53.31 vs. PIF 41.05; p=0.032, Wilcoxon-Mann-Whitney test). The improved clinical score persisted for 20 days post-therapy and remarkably even after 70 days from inoculation the protective effect of PIF remained significant.

In order to determine whether a higher PIF dose would further ameliorate disease course, mice were treated intermittently on days 5-27 and then, on days 44-62- with PIF (1.5 mg/Kg) or with vehicle only, while continuing to monitor the EAE course. (Figure 1C) The PIF treated mice had a significantly milder disease score (ATSD: PBS 39.79 vs. PIF 34.92; p=0.026) than control mice. However, the increased PIF dose did not further improve ATDS versus the lower dose, confirming that the optimal effects are obtained in a physiologic concentration [1]. Overall data reveal that PIF is effective in reducing paralysis in a clinically realistic acute MS model long-term.

### PIF promotes brain re-myelination

**Figure 2:**
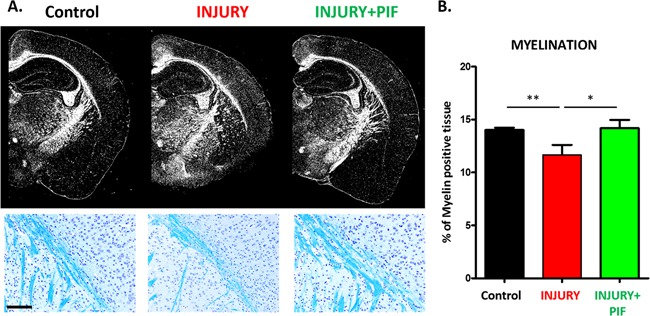
PIF promotes brain re-myelination PIF effect on myelin expression was compared to vehicle treated control and naïve SJL mice. Briefly, at day 28-30 after RR-EAE induction (control n=3; Injury n=5; Injury+PIF n=8) mice fixed brains were embedded in paraffin and sectioned into 7μm slices. Slides were stained in Cresyl violet (Nissl body staining for neuronal structure and gross brain morphology) and Luxol Fast Blue revealing areas of myelination in the subcortical white matter. **(A)** Representative images of subcortical white matter comparing the three groups. **Upper panel** shows the brain morphology and myelination. PIF treated injured brains (Injury+PIF) are similar to naïve mice (control) and vehicle treated injured brains (Injury) show impaired myelination in the subcortical region. **Lower panels**: Representative images of the subcortical region (blue staining: luxol fast blue) display PIF`s induced myelination (compare Injury+PIF versus Injury and Injury versus Control). **(B)** Quantitative analysis of myelin positive cells in the groups. PIF treatment results in restored myelination in the brain. *p<0.05 and ** p<0.01. PIF: PreImplantation Factor; scale bar: 100μm

Given that PIF ameliorates RR-EAE (Figure 1A–1C) and that the myelination deficit is a hallmark of MS, next we tested PIF effects on myelin expression. Indeed, the exposure to the M. smegmatis challenge (injury) resulted in a significant reduction of myelin positive cells (Figure 2A and 2B: compare Injury versus Control: naïve mice), confirming the previously reported observations [43]. Importantly, PIF treatment resulted in significant amelioration of the induced myelination loss (Figure 2A and 2B: compares Injury+PIF versus Injury alone). The results in the PIF treatment group are similar to that observed in controls (Figure 2A and 2B: compare Injury+PIF to Control). We hypothesize that the observed effect on brain myelination and the previously reported effect on spinal cord myelination [28] are mainly due to modulation of the inflammatory responses. Thus, whether PIF directly targets the brain to promote re-myelination was tested next.

.

### PIF crosses the BBB and targets brain microglia

**Figure 3:**
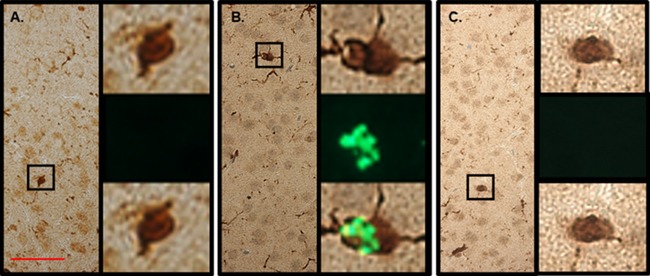
PIF targets microglia in the brain SJL mice previously infected with rMSp139 and in late phase of chronic disease (> 60 days after infection) treated with PIF or PBS for 3 weeks were injected with a single FITC-PIF or PBS dose and sacrificed 3 hours later. We used FITC-scrambled PIF as negative control as well. Brains were prepared for histology. **(A)** In the PBS injected mice the Iba-1 positive cells (microglia) showed predominantly amoeboid state and negative PIF staining. Right panels show Iba-1 positive cell (upper), negative PIF immunofluorescence (middle) and merged images (lower). **(B)** In PIF treated animals microglia was predominantly in ramified state and PIF positive. Right panels show Iba-1 positive cell (upper), positive PIF immunofluorescence (middle) and merged images (lower). **(C)** In the PBS injected mice with FITC-scrambled PIF (negative control) the Iba-1 positive cells (microglia) showed predominantly amoeboid state and expectantly negative PIF staining. Right panels show Iba-1 positive cell (upper), negative PIF immunofluorescence (middle) and merged images (lower). Scale bar 50 μm.

It was previously shown that following brain trauma PIF crosses the BBB to target microglia and neurons [26]. To assess whether in chronic RR-EAE PIF can also cross the BBB to reach the CNS was determined next. 11 week old female SJL mice, previously infected with rMSp139, and at a chronic phase of disease (>62 days after infection) treated with PIF or PBS for 3 weeks were injected with a single dose of FITC-PIF, PBS, and scrambled-PIF-FITC (inactive peptide). In the PBS injected mice (Figure 3A) the Iba-1 positive cells (microglia) showed predominantly amoeboid state and negative PIF staining. In line with previous reports [26], in PIF treated animals microglia was predominantly in ramified state and PIF positive (Figure 3B). Scrambled PIF-FITC was PIF negative (Figure 3C). Thus we conclude that PIF crosses the BBB and targets the brain during the chronic inflammation phase. These results support PIF use for targeted therapy in MS.

### PIF reduces pro-inflammatory cytokines in lymph node while not altering T-cell recruitment

**Figure 4:**
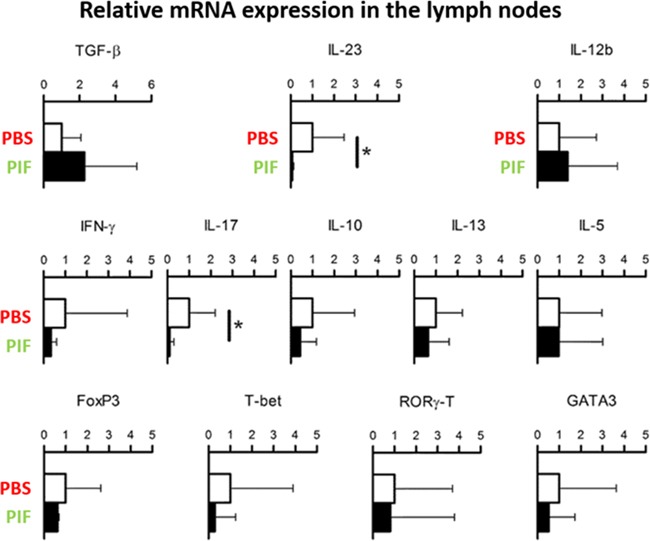
PIF modulates cytokine expression in draining lymph nodes SJL mice (5 each group) were infected with rMSp139 and treated daily with 0.75 mg/Kg of PIF (**black bars**) or vehicle only (**white bars**). Ten days later, cells from draining lymph nodes were obtained and cultured for 3 hours. Levels of mRNA specific for the indicated cytokines and transcription factors were measured by quantitative RT-PCR. PIF reduced the expression of IL-17 and IL23 significantly. *p<0.05 (Mann-Whitney Test); PIF: PreImplantation Factor.

Neuroinflammation is a progressive process where in early stages the innate immune system is the main participant but as inflammation progresses the adaptive arm of immunity becomes functional. Therefore, we initially tested the effect of PIF in early-stage disease where systemic cytokines may be affected. Given that the systemic effects of PIF are well described [24] [28], we tested the gene expression of crucial cytokines (acute time point of the injury at day 10) (Figure 4). Indeed, we detected significantly reduced Interleukin (IL)-23 and IL-17a cytokine expression in the lymph nodes. Notably, IL-23 is part of the innate immunity (produced mainly by dendritic cells and macrophages) and promotes the expansion of CD4+ T helper (Th)-cells secreting IL-17 (Th17) which is a potent pro-inflammatory cytokine [47] IL-17 plays a dominant role in MS (and EAE) and in several other autoimmune diseases [48]. Further, the ability of PIF to modulate IL-23 while preserving IL-12B expression (which regulates polarization of T-cells to Th1 phenotype) may explain pregnancy-induced protection against autoimmunity concurrent with preservation of anti-pathogenic responses.

**Figure 5:**
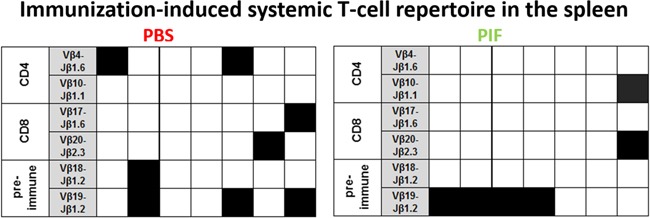
PIF does not modulate splenic T cell repertoire SJL mice (7 each group) were infected sc with rMSp139 and treated daily with 0.75 mg/Kg PIF or vehicle (PBS). Thirty days later, cells from spleen were obtained and cultured in the presence or absence of p139. mRNA was obtained and submitted to TCR BV-BJ spectratyping for the shared rearrangements characterizing the induced CD4+ T cells specific for p139 (Vb 4-Jb1.6; Vb10-Jb1.1), the T cells spontaneously responding to this epitope (Vb18-Jb1.2; Vb19-Jb1.2), and the induced CD8+ T cells specific for p139 (Vb17-Jb1.6; Vb20-Jb2.3). Each column reports data from one individual mouse, and a black square indicates the detection of T cells bearing the indicated TCR rearrangement. TCR: T cell repertoire; PIF: PreImplantation Factor.

The adaptive arm of immunity may be affected in RR-EAE as well. Therefore, we examined the effect of PIF or PBS injections administered until day 30 of the study. Following sacrifice isolated spleen cells were cultured and activated by antigen PLP-p139 or control. We aimed to characterize the immunization-induced systemic T-cell repertoire epitopes involved in MS: 1. CD4+ cells were characterized by the (Vb 4-Jb1.6; Vb10-Jb1.1) TCR rearrangements [42]. 2. The CD8+ cells were characterized by the (Vb17-Jb1.6; Vb20-Jb2.3) rearrangements [43] specific for PLP-p139. 3. We also analyzed the T-cell repertoire present in spontaneously activated naïve mice (Vb18-Jb1.2; Vb19-Jb1.2 rearrangements) [48]. Figure 5 summarizes that PIF did not modify T-cells recruitment as compared with PBS treated mice. We hypothesize that PIF does not affect the systemic T cell lineage to maintain the anti-pathogenic activity of cells that do not access the CNS and therefore are not pathogenic for development of RR-EAE.

### PIF regulates genes involved in solute transport, oxidative stress, and protein degradation in the brain

**Figure 6:**
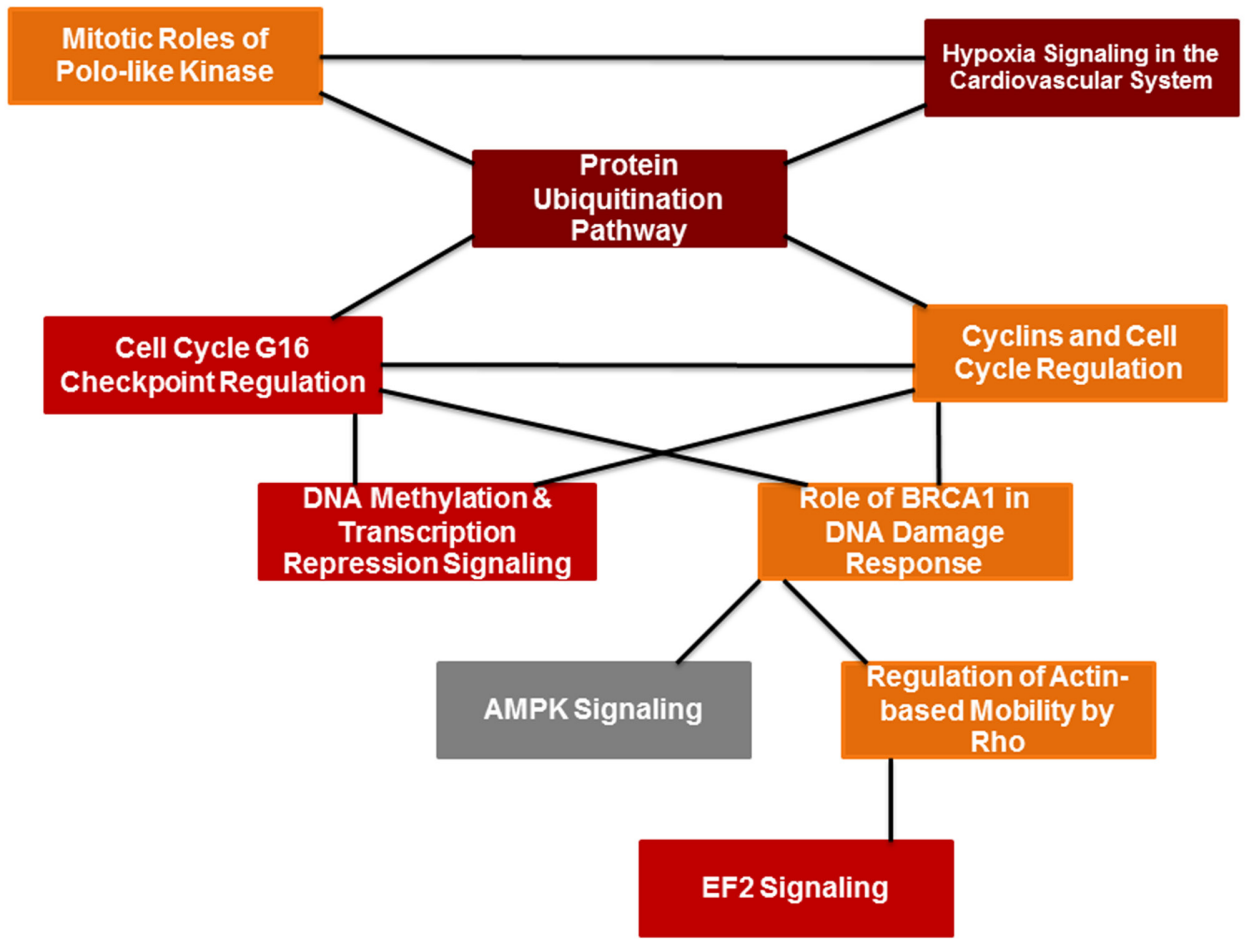
PIF effect of global brain genome pathways (Ingenuity analysis) Schematic pathways that are significantly affected by PIF and their interaction. Leading among them was the ubiquitin pathway that is responsible for protein degradation which was down-regulated by PIF. This was coupled with reduction in hypoxia signaling that creates vascular inflammation thereby the oxidative stress is reduced as well. On the other hand, the EIF2 signaling pathway increased which is responsible for the promotion of protein synthesis.

**Figure 7:**
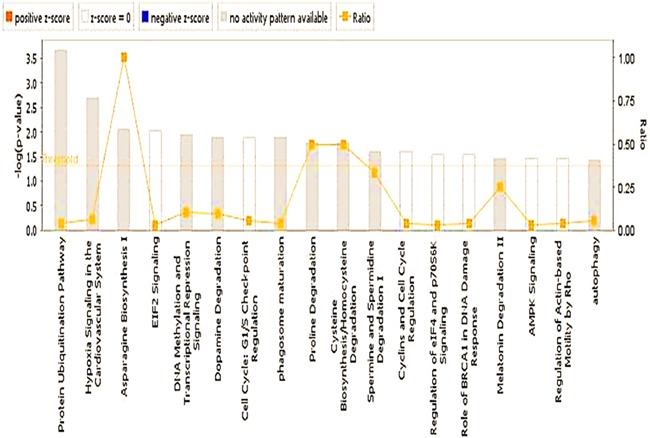
PIF effect on global genome (Ingenuity statistics) Evaluation of the pathways involved describing the effect PIF whether it is up or down regulated as well the associated level of significance. Namely the reduction in ubiquitination and EIF2 signaling were the most pronounced. On the other hand, the most remarkable increase was present in asparagine biosynthesis which protects against vascular damage.

In the classic EAE model we found that PIF had a major regulatory effect on the spinal cord genome and proteome [28]. Since in this study PIF led to restored myelination and reduced paralysis we examined the effect on the global brain gene expression as well. We detected a total of 168 genes which expression were increased or decreased by PIF as compared with the PBS treated group (p<0.05 (Supplementary Table 1). Ingenuity-based pathway analysis demonstrated that effect by PIF on neuronal disorders had the highest ranking as determined by the Z score (Table 1). This was followed by a significant effect on movement disorders and paralysis which supports our results (Figure 1, 2, 3). Detailed pathway analysis (Figure 6 and 7) showed that PIF modulates multiple genes involved in hypoxia and protein degradation induced by ubiquitination. PIF induced effects are visualized in a heatmap analysis as well (Supplementary Figure 2). The data revealed that PIF affected pathways involved in protein formation, EF4A1– RNA binding and degradation through Rnf13 (E3 Ubiquitin-Protein Ligase) pathway. The largest group of genes identified known to be critical for brain function are several solute carriers (SLC7a10, SLC2a1, SLC25a11, SLC7a14, and SLC24a3) involved in Na+/K+/Ca++ ions exchange, while others regulate glucose and amino acid transport.

**Table 1:** PIF protects against brain infection/inflammation: genome Z score analysis (Ingenuity)

CONDITION	Z log
Neuromuscular disease	3.209
Movement disorders	3.095
Progressive motor Neuropathy	3.02
Disorders of basal ganglia	2.62

Ranking is based on the score that determines the highest association within the pathways among the gene clusters. This was determined by using the 168 genes that up or down regulated (Supplementary Table 1). When the top disease was ranked 40 different genes were associated followed by 30 genes regulated by movement disorders: skeletal and muscular. Further data provided in Supplementary Table 2.

The global gene analysis further revealed that PIF reduces several genes involved in oxidative stress and protein misfolding (Figure 6, Supplementary Tables 1, 2). The reduced heat shock proteins and ubiquitination genes (CUL1, UBE2E1, UBE2Q1, PSMD1, HSP40, HSP90AB1, SUMO1) was coupled with (USP54, an ubiquitin peptidase) up-regulation which may protect against protein degradation [49]. PIF also regulated EIF2 eukaryotic initiation factor genes (EIF2B1, EIF3I, EIF4A1, PPP1CB, RPL22) to potentially reduce cellular stress. This pathway also initiates methionyl-tRNA and mRNA transfer to the 40S ribosomal subunit forming the 48S complex required for effective protein synthesis [49]. Notably, PIF also regulates aspargine biosynthesis pathway, which is altered in cases of microcephaly and is associated with progressive encephalopathy [50]. Among top ranking genes (Supplementary Table 1) PIF promoted spermine oxidase expression, where the encoded protein improves neurotransmission via cell-surface receptor and regulates reactive oxygen species [51]. PIF also promoted StAR expression involved in acute regulation of steroid hormone synthesis converting cholesterol to pregnenolone. In the brain StAR is restricted to specific neurons where it has neuroprotective effects. Finally, increased ARRB2, arrestin beta promotes synaptic receptors and MCF2L axonal transport in the brain [52]. Supplementary Figure 3 shows a comprehensive analysis of PIF induced action-based on Ingenuity program. Expectantly, PIF modulates multiple genes involved in neurologic diseases, skeletal and muscle disorders, and Cancer disorders [22–24, 26, 27]. Multiple molecular and cellular functions and physiological and system development and function genes are modulated by PIF as well. In line with the notion of PIF as essential pregnancy peptide embryonic development and DNA replication, recombination, and repair genes are top ranked [1]. Detailed analysis is presented in Supplementary Tables 1 and 2. Overall the gene data reveal potential mechanisms involved in PIF induced neuroprotection.

### PIF-induced upstream regulation involves MYCN (ERK/MAPK signaling) and cortisol binding site (NR3C1)

MYCN- neuroblastoma homolog gene regulates cell proliferation, survival, and apoptosis: (Supplementary Table 2) This upstream regulator involved in the myc signaling and is regulated by let-7 microRNA. Let-7 is down-regulated in the brain by PIF, thereby protecting against hypoxic ischemic brain damage [26]. PIF increased both (RPL22, SLC2A1) genes expression. Since these genes are upregulated by MYCN, indicates that this pathway is also regulated by PIF. In contrast, PIF decreased the expression of (NCL, E2F5, HSPAB1, EIF4A1, RBBP7, TPI1, ACTB) genes. Since MYCN activates (NCL, E2F5, HSP90AB1, and EIF4A1) genes indicates that PIF down-regulates MYCN effect. Finally, in case of ACTB both MYCN and PIF act in a similar manner to down-regulate the same gene. Also MYCN targets NCL a gene which expression is decreased by PIF. The second ranking upstream regulator is NR3C1 a glucocorticoid receptor that plays a major role in cell proliferation, remodeling and apoptosis: NR3C1 is a homodimer that interacts with HSP90, a PIF target [22]. PIF Increased the expression of: (USP54, BRD2, ARRB2) and decreased the expression of (WDR37, CUL1, SIAHA, HIC2. HSP90AB1, ATG12, PTP4A2, TM2D2, NMT1, ABI1, ACTB) genes. NR3C1 also interacts with SUMO1, an ubiquitin related gene which expression is decreased by PIF. Overall this data implies PIF involvement in MYC as well as glucocorticoid signaling, reflecting regulation of inflammatory response in the brain.

### PIF promotes BDNF, SLC2A1, and reduces HSP90AB1, and E2F5 genes expression in primary astrocytes

**Figure 8:**
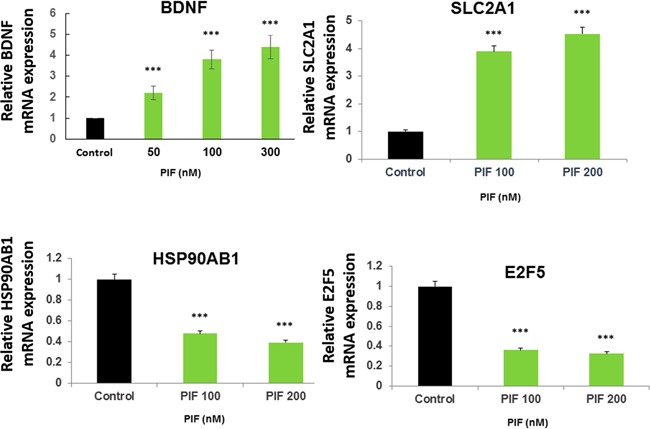
PIF promotes BDNF, SLC2A1, and reduces HSP90AB1, and E2F5 expression in astrocytes Primary astrocytes were cultured with increasing PIF concentrations up to 48 hours. PIF promotes BDNF expression (myelin synthesis inducer), SLC2A1 expression (glucose transporter) while reducing HSP90AB1 expression (oxidative stress), and E2F5 expression (neuro-injury activated). mRNA levels were determined using RT-PCR of the specific genes compared to S12 expression. For each factor the control levels were set as 1 and the expression of the treated samples was compared to the control. *** p<0.001. PIF: PreImplantation Factor.

Originally astrocytes were viewed as support cells for the brain, however, as recently indicated they play a major role in CNS microarchitecture and neural cells development. Also as recently shown these cells greatly facilitate interneuron communication [53]. Moreover, astrocytes protect the brain by releasing cytokines and neurotrophic factors; importantly, the brain derived neurotrophic factor (BDNF) [54]. These cells through BDNF action were shown to restore brain myelination in a neuroinflammatory disease model [55]. We found that PIF promoted the expression of BDNF by cultured primary astrocytes in a dose dependent manner (Figure 8). Therefore the observed effect on brain re-myelination (Figure 3) is partially exerted by PIF targeting brain astrocytes which are associated with increased BDNF expression. Whether the effect of PIF on brain genes expression (Supplementary Tables 1 and 2) is exerted by targeting local astrocytes was examined next. PIF regulates several brain solute transporters including SLC2A1 which is involved in glucose transport. We tested PIF effect in primary astrocytes cell culture documenting a dose dependent increase in SLC2A1 gene expression, thus confirming the brain genome data (Figure 8). In contrast, PIF reduced HSP90AB1 expression preventing protein degradation. Notably the protein product is a major PIF target [20, 22]. PIF also reduced E2F5 expression (a cell cycle protein) (Figure 8). This gene is expressed in the spinal cord following post-traumatic injury [56]. Thus PIF may regulate the expression of genes in the brain by targeting local astrocytes.

### PIF promotes IL10 and reduces IFNγ expression by primary microglia cultures

**Figure 9:**
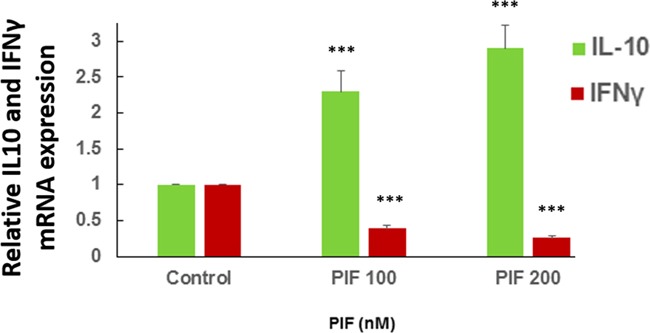
PIF promotes IL10 and reduces IFNγ expression in microglia cultures Primary microglial cells were cultured with increasing PIF concentrations up to 48 hours. PIF increased anti-inflammatory IL10 while reducing pro-inflammatory IFNγ expression in a dose dependent manner. mRNA levels were determined using RT-PCR of the specific genes compared to S12 expression. For each factor the control levels were set as 1 and the expression of the treated samples was compared to the control *** p<0.001. PIF: PreImplantation Factor.

In the current study PIF was shown to directly target microglia (Figure 3). Whether PIF also regulates these brain derived primary macrophages was tested next. In microglia cell lines PIF promotes IL10 [26, 27]. Herein the effect of PIF was tested on primary mouse cells examining effect on IL10 and Interferon (IFN)γ cytokines secretion (Figure 9). Data documented the dual action of PIF as PIF increased the pro-tolerance IL10 while reducing the pro-inflammatory cytokine IFNγ secretion. Overall the cell based data support PIF's direct effect on the brain.

## DISCUSSION

There is an urgent need to develop safe, easy to administer and effective MS/neuroinflammation therapy. Due to the sub-clinical and indolent nature of progressive neurologic disorders they are especially difficult to diagnose early and treat. In the MS brain the loss of myelin is key evidence for disease progression therefore safe reversal would be a major advance. Our major finding is that PIF targets brain microglia to promote re-myelination in a post-infectious clinically realistic RR-EAE model. Addressing critical aspects of the disease through an integrated action PIF lowered the paralysis score while reducing systemic inflammation. Global brain gene analysis revealed that PIF promoted expression of Na+/K+/Ca++, glucose and amino acid transporters, while reducing oxidative stress and protein degradation. In primary astrocytes culture PIF promoted BDNF expression – key for myelin synthesis and in primary microglia increased IL10 while reducing IFNγ cytokines secretion. Together our data support PIF testing in a clinical setting for treatment of MS.

MS has variable clinical course and tends to be progressive. As PIF is effective long-term when administration is either continuous or episodic, it supports the clinical potential. PIF therapy in early stage of the disease or even when progressive may be envisaged. Since intermittent PIF administration led to reduced paralysis which lasted ∼80 days- it supports potential long-term efficacy, perhaps for years in patients. A similar long-term protection was found using PIF in the classic EAE model [28]. In our study a single daily, low physiologic PIF regimen was found to be effective. Such minimally invasive dosing is expected to increase patients’ compliance. Collectively, PIF protects against neuroinflammation long-term irrespective if it is antigen driven or is of infectious origin.

Although infection by the Smegmatis bacteria combined with PLP activates the immune system, these bacteria are rapidly eliminated while the inflammatory response remains. As such the post-infectious- inflammatory EAE (RR-EAE) replicates clinical MS features as shown by the significant demyelination of the brain. Remarkably, PIF restored myelination in the brain. This key finding addresses a pathology which is difficult to achieve by current therapy especially when MS symptoms become progressive. In our RR-EAE model the focus was the brain and finding that PIF directly targets the microglia (immune element) is crucial. This is highly relevant since PIF was administered during the chronic phase of the disease and apparently at that point the BBB was intact. On the other hand, current drugs used for the treatment of neurologic diseases have to be of small size or lipid soluble in order to cross by trans-membrane diffusion. The use of transporters may improve larger proteins and peptides access to the CNS [57]. In the hypoxic-ischemic encephalopathy model PIF also directly targets brain microglia and neurons while is rapidly cleared from the circulation [26, 27]. Thus PIF directly targets the brain which leads to re-myelination and reduced paralysis.

Immune disorders, including neuroinflammation/MS have both local and systemic components. In order to prevent relapse, they both have to be addressed. PIF directly targets the systemic immunity *in vitro* and these observations were confirmed also *in vivo* [17, 19, 20, 23, 28]. The lower IL-23 and IL-17 cytokines expression in lymph nodes reflect systemic protection. Similar to the IL-23 dimer circulating IL-12 levels were reduced by PIF in the classic EAE model preventing paralysis development [28]. PIF targets macrophages which secrete IL-23 and IL-12 cytokines [24] [58]. IL-17 plays a key role in autoimmunity, and PIF reduced this pro-inflammatory cytokine levels in PLP-activated splenocytes [28]. Interestingly, the T-cell repertoire recruitment was not affected by PIF. Thus, the innate (at the acute phase at day 10) but not the adaptive arm of immunity (at sub-acute phase at day 30) are affected by PIF. Such duality of action preserves the beneficial anti-pathogenic effect. The relative importance of PIF induced systemic as compared with CNS protection cannot be determined at present and will be addressed in future studies. Overall PIF's integrated systemic and/or central protection leads to amelioration of the clinical score.

In classic EAE contrary to our current RR-EAE model, the focus was the spinal cord while in MS brain inflammation is dominant. Mechanistically in classic EAE in the spinal cord PIF reduced oxidative stress and inflammatory response while promoting neural repair by reduced tubulin breakdown and increased axon assembly. The neuroregenerative effect was coupled with improved synaptic transmission [28]. For the first time using this post-infectious, clinically relevant RR-EAE model a given drug therapy was tested. The effect of PIF on the global brain genome was evaluated by two independent complementary methods of analysis. In this model the damage is caused by the ensuing inflammatory response to a transient exposure to bacteria. Thus this model likely represents the MS pathology. In classic, EAE neuroinflammation practically is only antigen driven. Interestingly, we find that PIF regulates both infection and inflammation induced genes expression. Irrespective whether the smegmatis bacteria is innocuous and promptly eliminated by the organism, its foot-print persists >70 days- evidenced by the resulting chronic inflammation.

PIF primarily affected genes are involved in reducing oxidative stress, cell cycle check-point regulation, and DNA methylation pathways – which support involvement in protective mechanisms. For example, by regulating the ubiquitination pathway PIF may prevent protein degradation - key for neurodegenerative diseases- i.e. prions. Effect on EIF2 related genes reflect amino acids that are processed post-mRNA activation supporting protein neo-synthesis. The reduction in pro-inflammatory CD4+ cells activation may further aid in the neuro-repair process. Interestingly, the highest ranking upstream regulator of PIF was MYCN and expression of microRNA let-7 members is controlled by MYC binding to their promoters. This is not surprising as let-7 is down-regulated by PIF [26]. Together, these results suggest that the PIF protective effects in RR-EAE model may involve similar protective mechanisms [26–28]. Notably, PIF affected genes associated with diverse neurologic disorders, beyond those involved in MS. This raised the possibility that similar or the same infectious factor can lead to progressive brain inflammation and degeneration. For example, increased RPH3A, Ras-related protein Rab-3A promotes synaptic vesicle traffic and fusion while aberrant interaction with alpha-synuclein leads to aggregates found in Huntington chorea [59]. SFRS6 constitutively splice/missplice Tau exon-10 causes fronto-temporal dementia [52]. VARS gene is involved in development of brain malformation [60]. SPG7, ATP-dependent zinc metalloprotease is involved in development of spastic paraplegia [61, 62] while BRD2 gene microdeletion is associated with juvenile myoclonic epilepsy development [22]. On the other hand the decreased expression of ATPase and ubiquitin genes (ATP6AP2, UBE2e1, and Ube2q1) prevent mitochondrial oxidative stress leading to Parkinson's disease, Alzheimer's and X-linked mental retardation. SPG21 is a negative pro-inflammatory CD4+ cells regulator that can lead to spastic paraplegia while the aberrant PDCD10 expression can be associated with the development of cavernous cerebral malformation [63]. Several other gene function role needs to be examined in future studies. Thus, beyond MS PIF may also protect against other inflammatory neurological disorders, this possibility is currently being examined.

Recently, astrocytes have been implicated in MS pathology and shown to play a key role in myelin synthesis [54]. We found that PIF promoted a key myelin synthesis inducer BDNF in primary astrocytes which support the potential interactions. Evidencing further astrocytes role in neural repair is shown by PIF promoting glucose transport (SLC2A1) while reducing oxidative stress related genes, confirming the gene array data. The presence of PIF- astrocyte interaction *in vivo* however this needs to be confirmed by further study. This is of special interest as astrocytes play an important role in blood flow regulation and since PIF negatively regulates phospholipase A2, activating protein (Plaa) expression (Supplementary Table 1) involvement in this important process is possible. We hypothesize that under basal O_2_ consumption PIF protects against oxidative stress [24] and phospholipase A2 promotes vasoconstriction. As O_2_ consumption rises, vasodilatation prevails and through Ca^++^ flux promotes arachidonic acid which via COX-1 action releases PGE2 [53]. Similarly, PIF also protected against aortic vascular inflammation by preventing macrophage induced atherosclerotic plaque formation [21]. Additional support for such hypothesis is the high canonical ranking of pathways where hypoxia signaling in the cardiovascular system was the second rank after protein ubiquitination. The second component is microglia as microglia play a key role in inflammatory response redirecting towards damage or repair depending on the activating agent [64]. In primary microglia cell cultures PIF promoted IL10 while reducing the major pro-inflammatory IFNγ expression providing an important dual protective effect [26]. Overall targeting primary astrocytes and microglia provides support for the observed neurotrophic effects of PIF in the CNS.

PIF protects against ionizing radiation induced pathologies locally and systemically where reduced oxidative stress and protein damage was evidenced [25]. Similarly PIF herein in the brain and recently in the spinal cord protects against oxidative stress and protein degradation [1, 28]. Brain cancer therapy beyond surgery requires radiotherapy which promotes inflammation and neurodegeneration. This supports the use of PIF in patients with brain cancer aiming to mitigate radiation induced damage, not affecting the beneficial anticancer effect, while promoting neurorepair [24]. Studies examining this hypothesis are currently ongoing.

We acknowledge that a minor flaw of our study is the lack of the uninfected control in the study. However, we demonstrated previously that control animals injected with a control plasmid strain rMSHA compared to rMSp139 (the active agent) did not cause paralysis [43]. The strengths of our study are the multiple dosage paradigm used and multiple layers of evidence demonstrating PIF`s protective effect.

## CONCLUSION

Continuous or intermittent PIF administration directly promotes re-myelination while reducing paralysis in clinically-realistic MS/neuroinflammation model. Promotion of BDNF by cultured astrocytes- support possible local myelin synthesis. Local CNS protection is coupled with reduced systemic inflammation. PIF induced brain Na+/K+/Ca++ ions, amino acid and glucose transport is coupled with reduced oxidative stress and protein degradation pathways. The physiological PIF dose used, is similar to endogenous maternal circulating levels where MS symptoms frequently improve [4]. Upon completion of the ongoing Phase Ib clinical trial for autoimmune disease, phase II clinical trial for PIF testing in neuroinflammation/MS is planned.

## MATERIALS AND METHODS

### Mice, peptides and mycobacterium strain

Eight week-old SJL female mice were purchased from Charles River (Calco, Italy) and kept in a conventional facility at “Università Cattolica del Sacro Cuore” in Rome. All experimental procedures involving animals were approved by the internal Ethical Committee and by the Italian Ministry for Health. Peptide 139–151 of proteolipid protein (p139, HSLGKWLGHPDKF) was purchased from PRIMM (Milan, Italy) and was >95% pure by HPLC, as determined by mass spectrometry. Mycobacterium smegmatis Bacteria expressing the chimeric protein containing the p139 fused with MPT64 (rMS^p139^) was obtained as previously described [43].

### Synthetic PIF (PIF)

PIF, MVRIKPGSANKPSDD, and fluorescein isothiocyanate-labelled PIF (FITC-PIF) was provided by Bio-Synthesis, Inc. (Lewisville, TX). FITC-scrambledPIF-GRVDPSNKSMPKDIA) Peptide identity was verified by matrix-assisted laser desorption/ionization time-of-flight (MALDI-TOF) mass spectrometry and amino-acid analysis, and the peptides were purified to >95% by HPLC, as documented by mass spectrometry.

### RR-EAE induction and clinical evaluation

SJL female mice, 8-10 weeks old, were infected subcutaneously (s.c.) in the back with 4×10^6^ CFU of rMS^p139^ in PBS 100 μl/mouse. Notably, in this Smegmatis model we demonstrated previously that control animals injected with a control plasmid strain rMSHA compared to rMSp139 (the active agent) did not cause paralysis [43]. Clinical signs of EAE were evaluated daily and in a blinded fashion according to the following scale 0-5: 0, no clinical score; 1, loss of tail tone; 2, weak hind leg paresis; 3, hind leg paresis; 4, complete paraplegia; and 5, death or moribund. Intermediate values were assigned for incomplete symptoms. Average total score of disease (ATSD) was calculated as the average of the sum of daily scores of each mouse. Area under the curve (AUC) at the peak,* was calculated using a score of 0.5 as baseline in composite experiments [28].

### PIF treatment

To test PIF effects we used two different treatment regimens. First we tested continuous administration: Mice were injected intraperitoneally (i.p.) with PIF (0.75 mg/kg or 1.5 mg/kg in PBS 100 μl daily) starting on day 3 after infection with rMS^p139^ until the end of the experiment. The control group received vehicle only. Next we tested intermittent administration: Mice were injected i.p. with PIF (0.75 mg/kg in PBS 100 μl daily) starting at symptom onset and continuing until remission of the symptoms. We resumed administration at subsequent relapse. Again, the control group received PBS vehicle only.

### FITC-PIF in the brain (cross intact BBB)

Mice, previously treated with continuous daily administration for 3 weeks with PIF 0.75 mg/kg, or PBS were tested. By day 62 after infection mice were treated with an injection of FITC-PIF 0.75 mg/kg in 100 μl of PBS or with vehicle only. An additional negative control FITC-scrambled PIF was injected as well. At 3 hours after injection mice were sacrificed and the brains were analyzed.

### Tissue harvesting and brain myelin evaluation

At day 28-30 after EAE induction, mice under deep anesthesia (Ketamine 75 μg/Kg, Medetomidine 1 μg/Kg i.p.) were perfused with 50 ml of PBS and sacrificed. Spleen and brain tissue was collected. Tissue was placed in nitrogen in cryovials for 10 min and stored at-80C. Given that myelin loss is a prominent event in MS we evaluated a myelin staining technique [45, 46]. Briefly, fixed brains were embedded in paraffin and sectioned into 7μm slices. Slides were rinsed in ddH2O, counterstained in Cresyl violet (Nissl body staining for neuronal structure and gross brain morphology) and Luxol Fast Blue (to reveal areas of myelination in the subcortical white matter), dehydrated in a series of ethanol baths (95% >100%) and xylene, and mounted with Eukitt (Sigma-Aldrich, St. Louis, MO). For FITC-PIF localization in the CNS, mice were perfused under deep anaesthesia (Ketamine 75 mg/Kg, Medetomidine 1 mg/Kg i.p.), through the aorta with 50 ml of PBS, followed by 50 ml of 4% paraformaldehyde (VWR international). Brain were removed and immersed in the same fixative for 24 hrs. Tissue blocks were routinely embedded in paraffin and 10 μm thick slices were prepared. Staining have been made automatically with the immunostainer Leica Bond RX. In brief, after deparaffinization of the slides, the target was retrieved in citrate buffer (10 mM; pH 6.0; Leica) for 30 min. A rabbit polyclonal antibody against the ionized calcium binding adaptor molecule 1 (Iba-1, Abcam, ab5076, 1:100), a highly specific marker for microglia, was used to localize both resting and activated microglia [26, 27]. Monoclonal antibody anti-PIF (1:100, GenWay, San Diego, Ca, USA) was used to detect sPIF. After quenching endogenous peroxidase activity, slides have been incubated 30min with anti-Iba1 antibody, followed by incubation with a rabbit anti-goat secondary antibody (DAKO, 1:400, E0466) for 15min. An anti-rabbit Poly-HRP IgG polymer was then applied to the slides for 15 min at RT, followed by application of DAB+ chromogen in buffer substrate for 10 min (Bond Polymer Refine Detection Kit). Additionally, PIF expression was detected using a fluorescent setup. After Iba1 staining, slides were blocked with 10% goat serum/1% bovine albumin/PBS followed by incubation with the anti-sPIF antibody for 1h at RT and then FITC-labeled streptavidin (1:200, Sigma-Aldrich) for 1h at RT. The tissue was finally counterstained with hematoxylin and slices were mounted with VECTASHIELD (Mounting Medium with DAPI). We acquired images using a BX51 microscope (Olympus, Tokyo, Japan) with a 20x or 40x objective and equipped with a digital camera. An independent observer acquired sections visual field by visual field without overlapping per hemisphere and animal for each specific immunostaining blinded to the experimental conditions.

### Cytokine production and transcription factor mRNA expression

Following immunization and PIF or vehicle administration, at day 10 of the experiment mice were sacrificed and the popliteal lymph nodes (LNs) were collected. Then the dissociated 5×10^6^ LN cells/well were cultured for 3 hrs in RPMI-1640 medium (Sigma- Aldrich, St Louis, MO, USA) supplemented with 2mM L-glutamine, 50 μM 2-ME, 50 μg/ml gentamicin (Sigma- Aldrich, St Louis, MO, USA) and 0.2% mouse serum. LN cells were then re-suspended in RLT buffer for RNA extraction. Total mRNA was isolated using RNeasy Mini Kit (Qiagen, Valencia, CA) and cDNA was synthesized using qScript™ cDNA SuperMix (Quanta BioSciences, Inc., Gaithersburg) according to the manufacturer's instructions. Quantification of mRNA was performed at 260 nm using NanoDrop 1000 (Thermo Scientific, Waltham, MA).

### Quantitative mRNA expression

qRT-PCRs were performed using iQ SYBR^®^ Green Supermix and an iQ5 Real-Time PCR Detection System (Bio-Rad, Hercules, CA). Relative expression levels of cytokine and transcription factor mRNAs were normalized using 18S as the housekeeping gene and calculated with the 2−ΔΔCt method. Samples were loaded in triplicate. qRT-PCR was followed by a melting curve to assess presence of specific replicons and primer dimers. The following primers (Invitrogen™ Life Technologies, Paisley, UK) were used: mouse Interferon (IFN)-γ, forward 5′-CAG CAA CAG CAA GGC GAA AAA GG-3′ and reverse 5′-TTT CCG CTT CCT GAG GCT GGA T-3′; mouse FoxP3, forward 5′-CCT GGT TGT GAG AAG GTC TTC G-3′ and reverse 5′-TGC TCC AGA GAC TGC ACC ACT T-3′; mouse Interleukin (IL)-6, forward 5′-ACA CAT GTT CTC TGG GAA ATC GT-3′ and reverse 5′-AAG TGC ATC ATC GTT GTT CAT ACA-3′; mouse IL-12b, forward 5′-GAA GCA CGG CAG CAG AAT-3′ and reverse 5′-AGC CAA CCA AGC AGA AGA CA-3′; mouse IL-13, forward 5′-AAC GGC AGC ATG GTA TGG AGT G-3′ and reverse 5′-TGG GTC CTG TAG ATG GCA TTG C-3′, mouse IL-17, forward 5′-CAG ACT ACC TCA ACC GTT CCA C-3′ and reverse 5′-TCC AGC TTT CCC TCC GCA TTG A-3′, mouse IL-23, forward 5′-CAT GGG CTA TCA GGG AGT A-3′ and reverse 5′-AAT AAT GTG CCC CGT ATC CA-3′, mouse Transforming Growth Factor (TGF)-beta, forward 5′-ACC CCC ACT GAT ACG CCT GA-3′ and reverse 5′-AGC AGT GAG CGC TGA ATC GAA-3′, mouse 18S, forward 5′-CTG CCC TAT CAA CTT TCG ATG G-3′ and reverse 5′-CCG TTT CTC AGG CTC CCT CTC-3′, mouse IL-10, forward 5′-GCT CCT AGA GCT GCG GAC T-3′ and reverse 5′-TGT TGT CCA GCT GGT CCT TT-3′, mouse IL-5, forward 5′-CTC TGT TGA CAA GCA ATG AGA CG T-3′ and reverse 5′-TCT TCA GTA TGT CTA GCC CCT G-3′, mouse t-bet (tbx21) forward 5′-AGC AAG GAC GGC GAA TGT T-3′and reverse 5′-GGG TGG ACA TAT AAG CGG TTC-3′, mouse RORγ-t forward 5′-CTA CTG AGG AGG ACA GGG AG-3′ and reverse 5′-AGT AGG CCA CAT TAC ACT GCT-3′, mouse GATA-3 forward 5′-CTC GGC CAT TCG TAC ATG GAA-3′ and reverse 5′-GGA TAC CTC TGC ACC GTA GC-3′.

### T cell receptor repertoire analysis

Repertoire analysis was performed using a modification of a described protocol [55]. For the TCR spleen repertoire analysis, 10^7^/well splenocytes were cultured on 24-well plates in RPMI-1640 medium (Sigma- Aldrich, St Louis, MO) supplemented with 2mM L-glutamine, 50 μM 2-ME, 50 μg/ml gentamicin (Sigma- Aldrich, St Louis, MO, USA) and 0.2% mouse serum in the presence or absence of 10 μg/ml p139. After 72 hrs splenocytes were re-suspended in RLT buffer for RNA extraction. Total mRNA was isolated using RNeasy Mini Kit (Qiagen, Hilden, Germany) and cDNA was synthesized using qScript™ cDNA SuperMix (Quanta BioSciences, Inc., Gaithersburg, MD) according to the manufacturer's instructions. Quantification of mRNA was performed at 260 nm using NanoDrop 1000 (Thermo Scientific, Waltham, MA). For the immunoscope analysis, cDNA was subjected to PCR amplification using a common constant β primer (Cβ 5′-CAC TGA TGT TCT GTG TGA CAG-3′) in combination with the variable β (Vβ) primer previously described [65]. Using 2 μl of this product as a template, run-off reactions were performed with a single internal fluorescent primer for each Jβ tested [65]. The products were then denatured in formalin and analysed on a 3130 Genetic Analyzer using Gene Mapper 4.0 (Applied Biosystem Foster City, CA, US). Results are reported as a relative stimulation index (RSI) [42], obtained from the ratio between the normalized peak area of cells stimulated with p139 and the normalized peak area of non-stimulated cells. T cells carrying a TCR rearrangement are considered expanded in a peptide-driven manner when RSI is > 2.

### Global gene expression

To detect the global gene changes in the brain we performed a gene array. Briefly, 30 mg of brain tissue (n=3 each, PIF versus PBS) was excised and homogenized in a Fastprep 120 tissue homogenizer (30 s at 4.0m/sec) in cell lysis buffer (Qiagen, Hombrechtikon, Switzerland). Total RNAs were extracted from cells using PureLink RNA Mini Kit (Ambion, Thermo- Fischer catalog number 12183018A). Total RNA (250ng) was amplified into cRNA using TotalPrep RNA amplification kit (AMIL1791, Ambion) following manufacturer's instructions. After amplification, 1.5 μg of cRNA was mixed with the hybridization controls and was hybridized to MouseRef-8 array (BD-202-0202, Illumina, USA). The array was hybridized for 16 hrs in a hybridization oven with a rocking platform at 58°C. The array chip then went through a series of washes before it was stained with streptavidin-Cy3. After the staining, it went through a final wash and drying. The array was scanned using the Illumina HiScan Scanner.

### Preparation and testing of PIF effects on of primary mouse astrocytes: Gene array validation

PIF's targeting of microglia in a cell line and neurons both *in vitro* and *in vivo* was demonstrated [26, 27]. However, whether PIF targets astrocytes, which emerged as an important cell type in neurodegenerative diseases such as MS, has not been tested [54]. Astrocyte cultures were prepared from 2-day old C57BL/6 mouse neonates. Cortices were isolated, stripped of their meninges and mechanically dissociated in ice-cold HBSS. The resulting cell suspension was then incubated with 0.05% trypsin for 25 min at 37° C followed by rinsing and filtration through a nylon mesh (70-μm pore size). The cells were then plated on collagen-coated plates and were maintained in astrocyte medium (ThermoFisher Scientific). Cells were studied after 2 weeks in culture. The effect of PIF 100 or 200nM on BDNF, recognized as key for MS therapy, was tested after a 48 hour culture [54]. In addition, three genes identified in the brain array were also validated using RT-qPCR, namely SLC2A1 (glucose transporter) and HSP90ab1 plus E2f5 related to oxidative stress and cell cycle control, respectively). At the end of the experiments cells were rinsed, then RNA was extracted and processed for RT-qPCR. Fold change was determined and compared to control. Data was generated in triplicate in three different experiments, setting significance at p<0.05.

### PIF effect on cytokine expression by primary microglia

Primary microglia were obtained from StemCells (Newark, Ca). These cells were isolated from 2 day old neonate C57BL/6 mouse brain tissues and placed in culture. The effect of PIF 0 to 200nM on IFNγ and IL10 expression was examined after 48 hours in culture. At the end of the experiments cells were rinsed, RNA was extracted and processed for qRT-PCR. Fold change was determined and compared to control. Data were generated in triplicate in 3 independent experiments, setting significance at p<0.05.

### Real-time quantitative PCR analysis of astrocytes and microglia

Total RNA was isolated from cultured astrocytes using QIAzol reagent (Qiagen, Valencia, CA) according to the manufacturer's protocol. 0.5 μg of RNA was employed to synthesize cDNA by Thermoscript (Invitrogen, Carlsbad, CA) with oligo dTprimers. A primer optimization step was performed for each set of primers to determine the optimal primer concentrations. Primers, 25 μL of 2x SYBR Green Master Mix (Invitrogen), and 30 to 100 ng cDNA samples were then re-suspended in a total volume of 50 μL PCR amplification solution. Reactions were run on an ABI Prism 7000 Sequence Detection System (Applied Biosystems, Foster City, CA). Cycle threshold (Ct) values were obtained from the ABI 7000 software. S12 or Δ-actin levels were also determined for each RNA sample as controls.

### Statistical analysis

Statistical analysis of the results was performed, when appropriate, with the two-tailed Wilcoxon-Mann-Whitney test for non-parametric values or with chi-squared tests, using GraphPad Prism 5.03 (GraphPad Software, Inc. La Jolla, USA); p<0.05 was considered significant. The output of the limma analysis was used to perform gene set enrichment analysis (GSEA) using the SetRank method. The key principle of this algorithm is that it discards gene sets that have initially been flagged as significant, if their significance is only due to the overlap with another gene set. It calculates the p-value of a gene set using the ranking of its genes in the ordered list of p-values as calculated by limma. The following databases were searched for significant gene sets: BIOCYC [66], Gene Ontology [67], ITFP [68], KEGG [69], LIPID MAPS [70], PhosphoSitePlus [51], REACTOME [62], and WikiPathways [71].

### Pathway ingenuity analysis

Genes found to be significantly different between PIF and control (p<0.05, two-tail Student's t test, n=168) were analyzed. We first determined the Z score to identify the highest association within the pathways. Further use of pathway analysis for ranking gene clusters and their association was accomplished by determining the statistical value of a pathway and whether the interaction led to up- or down-regulation of a given gene cluster.

## SUPPLEMENTARY MATERIALS FIGURES AND TABLES






