# Supplementary material for: PIF* promotes brain re-myelination locally while regulating systemic inflammation- clinically relevant multiple sclerosis M.smegmatis model
Source: Oncotarget. 2017 Feb 24;8(13):21834–51. doi: 10.18632/oncotarget.15662 (PMC5400627; doi:10.18632/oncotarget.15662)
Supplement: Supplementary file 1 [file oncotarget-08-21834-s001.pdf]

PIF* promotes brain re-myelination locally while regulating systemic inflammation- clinically relevant multiple sclerosis *M.smegmatis* model

SUPPLEMENTARY FIGURES AND TABLES

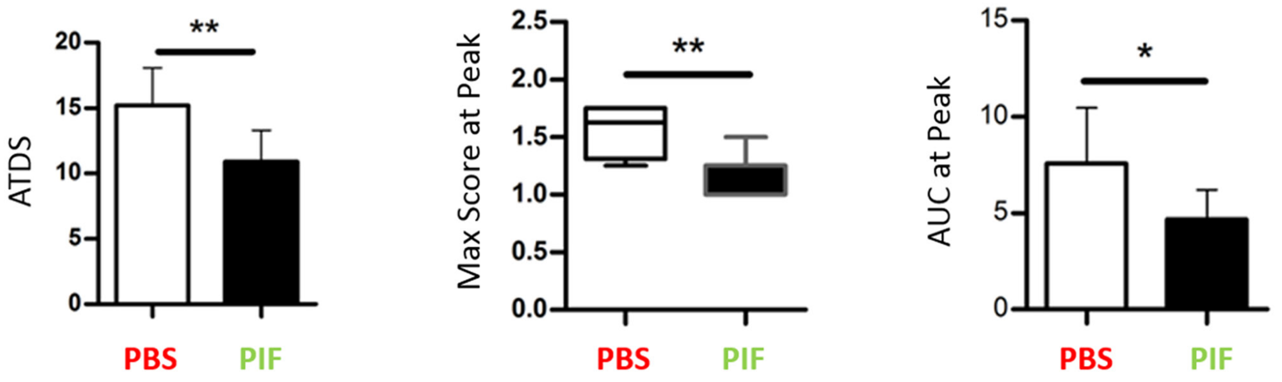

Supplementary Figure 1: Cumulative effect of PIF administration on the first acute episode of EAE until day 28 after infection. SJL mice were infected rMSp139 and treated daily with PIF or vehicle only (see Figure 1). Mice were treated after infection with 0.75 mg/Kg of PIF (n=6) or with vehicle only (n=4). **(A)** Average total score of disease, **(B)** maximum values of score at peak, and **(C)** the area under the curve of the peak of disease. Disease score was monitored by two independent examiners, blinded with respect to treatment. *p< 0.05; **p< 0.01 (Mann-Whitney test). PIF: PreImplantation Factor, ATDS: average total disease score; AUC: area under the curve.

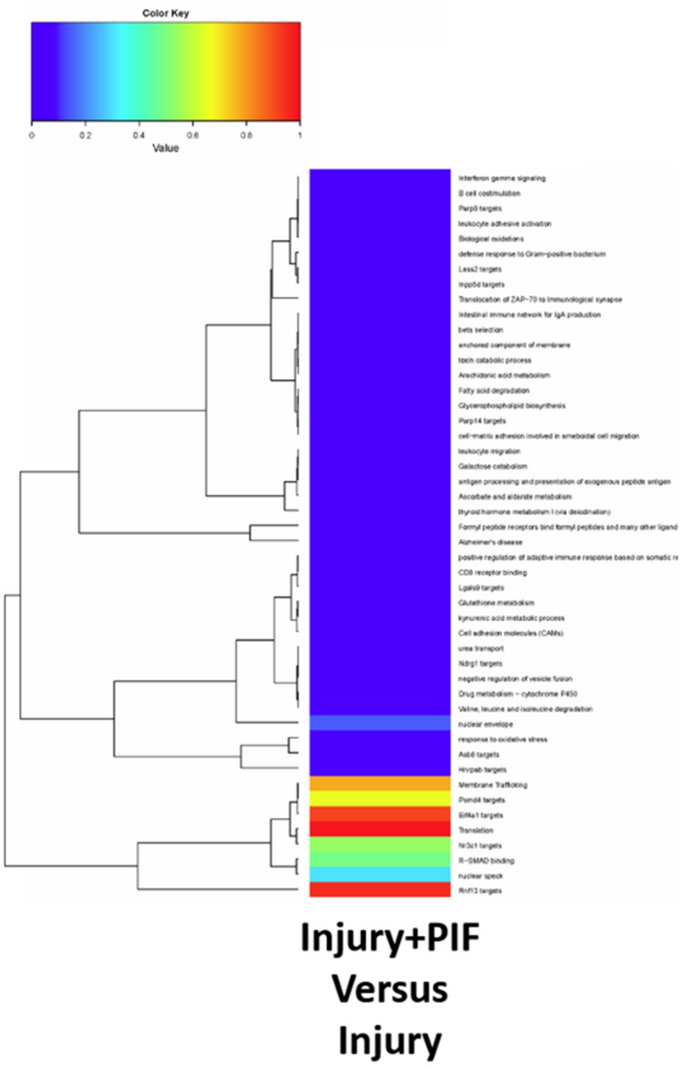

Supplementary Figure 2: Heatmap analysis of global brain genome. Pathways analysis results of the RNA array data. The color scale indicates the relative rank of pathway in a given comparison, with 1.0 meaning top rank and 0.0 indicating a pathway that was not found significant at all. Gene sets for brain injury of the different treatments (PIF versus PBS). Multiple pathways were affected such as protein formation and degradation involved in EF4A1-RNA binding and translation of proteins formed and ultimately degradation by Rnf13 (E3 Ubiquitin-Protein Ligase) pathway. Specific information listed in Supplementary Table 1.

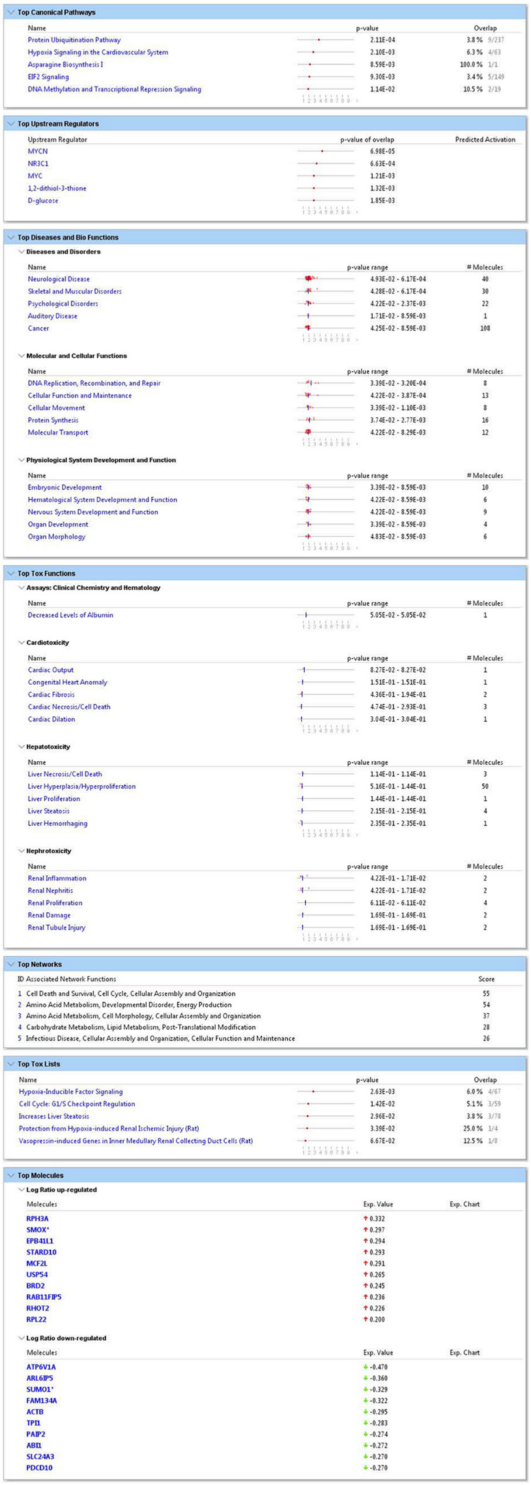

Top Canonical Pathways

Name	p-value	Overlap
Protein Ubiquitination Pathway	2.11E-04	3.8 % 9/237
Hypoxia Signaling in the Cardiovascular System	2.10E-03	6.3 % 4/63
Asparagine Biosynthesis I	8.59E-03	100.0 % 1/1
EIF2 Signaling	9.30E-03	3.4 % 5/149
DNA Methylation and Transcriptional Repression Signaling	1.14E-02	10.5 % 2/19

Top Upstream Regulators

Upstream Regulator	p-value of overlap	Predicted Activation
MYCN	6.98E-05	
NR3C1	6.63E-04	
MYC	1.21E-03	
1,2-dithiol-3-thione	1.32E-03	
D-glucose	1.85E-03	

Top Diseases and Bio Functions

Diseases and Disorders

Name	p-value range	# Molecules
Neurological Disease	4.93E-02 - 6.17E-04	40
Skeletal and Muscular Disorders	4.28E-02 - 6.17E-04	30
Psychological Disorders	4.22E-02 - 2.37E-03	22
Auditory Disease	1.71E-02 - 8.59E-03	1
Cancer	4.25E-02 - 8.59E-03	108

Molecular and Cellular Functions

Name	p-value range	# Molecules
DNA Replication, Recombination, and Repair	3.39E-02 - 3.20E-04	8
Cellular Function and Maintenance	4.22E-02 - 3.87E-04	13
Cellular Movement	3.39E-02 - 1.10E-03	8
Protein Synthesis	3.74E-02 - 2.77E-03	16
Molecular Transport	4.22E-02 - 8.29E-03	12

Physiological System Development and Function

Name	p-value range	# Molecules
Embryonic Development	3.39E-02 - 8.59E-03	10
Hematological System Development and Function	4.22E-02 - 8.59E-03	6
Nervous System Development and Function	4.22E-02 - 8.59E-03	9
Organ Development	3.39E-02 - 8.59E-03	4
Organ Morphology	4.83E-02 - 8.59E-03	6

Top Tox Functions

Assays: Clinical Chemistry and Hematology

Name	p-value range	# Molecules
Decreased Levels of Albumin	5.05E-02 - 5.05E-02	1

Cardiotoxicity

Name	p-value range	# Molecules
Cardiac Output	8.27E-02 - 8.27E-02	1
Congenital Heart Anomaly	1.51E-01 - 1.51E-01	1
Cardiac Fibrosis	4.36E-01 - 1.94E-01	2
Cardiac Necrosis/Cell Death	4.74E-01 - 2.93E-01	3
Cardiac Dilation	3.04E-01 - 3.04E-01	1

Hepatotoxicity

Name	p-value range	# Molecules
Liver Necrosis/Cell Death	1.14E-01 - 1.14E-01	3
Liver Hyperplasia/Hyperproliferation	5.16E-01 - 1.44E-01	50
Liver Proliferation	1.44E-01 - 1.44E-01	1
Liver Steatosis	2.15E-01 - 2.15E-01	4
Liver Hemorrhaging	2.35E-01 - 2.35E-01	1

Nephrotoxicity

Name	p-value range	# Molecules
Renal Inflammation	4.22E-01 - 1.71E-02	2
Renal Nephritis	4.22E-01 - 1.71E-02	2
Renal Proliferation	6.11E-02 - 6.11E-02	4
Renal Damage	1.69E-01 - 1.69E-01	2
Renal Tubule Injury	1.69E-01 - 1.69E-01	2

Top Networks

ID	Associated Network Functions	Score
1	Cell Death and Survival, Cell Cycle, Cellular Assembly and Organization	55
2	Amino Acid Metabolism, Developmental Disorder, Energy Production	54
3	Amino Acid Metabolism, Cell Morphology, Cellular Assembly and Organization	37
4	Carbohydrate Metabolism, Lipid Metabolism, Post-Translational Modification	28
5	Infectious Disease, Cellular Assembly and Organization, Cellular Function and Maintenance	26

Top Tox Lists

Name	p-value	Overlap
Hypoxia-Inducible Factor Signaling	2.63E-03	6.0 % 4/67
Cell Cycle: G1/S Checkpoint Regulation	1.42E-02	5.1 % 3/59
Increases Liver Steatosis	2.96E-02	3.8 % 3/78
Protection from Hypoxia-induced Renal Ischemic Injury (Rat)	3.39E-02	25.0 % 1/4
Vasopressin-induced Genes in Inner Medullary Renal Collecting Duct Cells (Rat)	6.67E-02	12.5 % 1/8

Top Molecules

Log Ratio up-regulated

Molecules	Exp. Value	Exp. Chart
RPH3A	↑ 0.332	
SMOX*	↑ 0.297	
EPB41L1	↑ 0.294	
STARD10	↑ 0.293	
MCF2L	↑ 0.291	
USP54	↑ 0.265	
BRD2	↑ 0.245	
RAB11FIP5	↑ 0.236	
RHOT2	↑ 0.226	
RPL22	↑ 0.200	

Log Ratio down-regulated

Molecules	Exp. Value	Exp. Chart
ATP6V1A	↓ -0.470	
ARL6IP5	↓ -0.360	
SUMO1*	↓ -0.329	
FAM134A	↓ -0.322	
ACTB	↓ -0.295	
TPI1	↓ -0.283	
PAIP2	↓ -0.274	
ABI1	↓ -0.272	
SLC24A3	↓ -0.270	
PDCD10	↓ -0.270	

Supplementary Figure 3: Function analysis of the gene network (Ingenuity) in the brain. PIF modulates multiple genes involved in neurologic diseases (40/168 genes), Skeletal and Muscle disorders (30/168 genes) and Cancer (108/168 genes). Multiple molecular and cellular functions and physiological and system development and function genes are modulated by PIF as well. In line with the notion of PIF as essential pregnancy peptide embryonic development and DNA replication, recombination, and repair genes are top ranked. Detailed analysis is presented in Supplementary Tables 1 and 2. Overall the gene data reveal potential mechanisms involved in PIF induced neuroprotection. PIF: PreImplantation Factor.

Supplementary Table 1: PIF effect on global gene analysis in the brain. We detected a total of 168 genes which expression were significantly increased or decreased by PIF as compared with the PBS treated group. PIF: PreImplantation Factor.

See Supplementary File 1

Supplementary Table 2: Gene network analysis: PIF affects genes involved in cell death and survival, amino acid metabolism and infection. Molecular and cellular function analysis revealed that PIF regulates genes involved in DNA replication and repair- reflecting a protective effect against abnormal protein formation and degradation. This is closely coupled with network function where the highest ranking was cell death and survival, followed closely by amino acid (protein) metabolism. (Supplementary Table 1). A total of 27/174 genes were affected. Among them, ATP6V1A, ACTB, SUMO1, and FAIM which is a FAS inhibitory factor (a death receptor-triggered apoptosis and regulates B-cells signaling and differentiation) were significantly down-regulated. Those involved in amino acid metabolism (26/174 genes) expression increased (BRD2, RPL22), while ARP6IP5 gene expression decreased. Amino acid morphology gene expression increased (RPH3A, MCF2L, RAB11FIP5, RPL22, BRD2), whereas (RB1, PAIP2) expression was down-regulated. Infection up-regulated genes were (USP54, RHOT2 Mitochondrial Rho GTPase) while, (ATP6V1A) expression decreased.

See Supplementary File 2
