# Supplementary material for: PIF* promotes brain re-myelination locally while regulating systemic inflammation- clinically relevant multiple sclerosis M.smegmatis model
Source: Oncotarget. 2017 Feb 24;8(13):21834–51. doi: 10.18632/oncotarget.15662 (PMC5400627; doi:10.18632/oncotarget.15662)
Supplement: Supplementary file 3 [file oncotarget-08-21834-s003.docx]

**Supplement Table IV: PIF Regulatory Pathways (Ingenuity)**

| **Cell Death & Survival** | | **Amino Acid Metabolism Energy** | | **Amino Acid Metabolism & Morphology** | | **Carbohydrate Metabolism** | | **Infectious Disease** | |
| --- | --- | --- | --- | --- | --- | --- | --- | --- | --- |
| **Up** | **Down** | **Up** | **Down** | **Up** | **Down** | **Up** | **Down** | **Up** | **Down** |
| CBS/LOC102724560  DPP9  EEF1B2  GGA1  MAPKAP2  SIN3A  SMARCC2  STIM1  TIMM44  VARS  WNK1  ZNF771 | ACTB  ATP6AP2  ATP6V1A  EIF4A1  FAIM  GABRG2  M6PR  MORF4L1  PLAA  PPID  RBBP7  SETX  SNUPN  SUMO1  ZHX1 | BRD2  CPSF1  HTRA1  LOC102724788/PRODH  PSMD1  RPL22  SLC2A1  SPHK2  WIPI2 | ARL6IP5  ATG12  CCDC59  CNTN1  DNAJB6  DNAJC6  EIF3I  GLRX3  MLYCD  NCL  PPP1CB  PSMD7  PTP4A2  SIAH1  SLC25A11  TERF2  YTHDF2 | AP3D1  ARRB2  GRIPAP1  KLC2  MCF2L  RAB11FIP5  RPH3A  STARD10  TRIB2 | ABI1  ASNS  BZW1  EIF2B1  HSP90AB1  MDH1  NDUFB9  PAIP2  PDCD10  STRAP  VAPA | BICD2  HBS1L  IFT172  JOSD2  MGAT1  NCAPH2  PIGQ  VPS16 | ADSS  DCTN5  DHODH  DHX32  GPM6A  LCMT1  PAPOLG  ZNF277 | CNOT3  CTDSP1  HS3ST3A1  POLG  RHOT2  USP54 | ATAD1  ATP6V0D1  ATP6V1A  ATXN10  CDC16  CHIC2  DOCK4  SPG21  UBE2Q1  ZC3H14 |

**Supplement Gene network analysis: PIF affects genes involved in cell death and survival, aminoacid metabolism and infection**

Molecular and cellular function analysis revealed that PIF regulates genes involved in DNA replication and repair- reflecting a protective effect against abnormal protein formation and degradation. This is closely coupled with network function where the highest ranking was cell death and survival, followed closely by amino acid (protein) metabolism. (**Table II supplement**). A total of 27/174 genes were affected. Among them, ATP6V1A, ACTB, SUMO1, and FAIM which is a FAS inhibitory factor (a death receptor-triggered apoptosis and regulates B-cells signalling and differentiation) were significantly down-regulated. Those involved in amino acid metabolism (26/174 genes) expression increased (BRD2, RPL22), while ARP6IP5 gene expression decreased. Amino acid morphology gene expression increased (RPH3A, MCF2L, RAB11FIP5, RPL22, BRD2), whereas (RB1, PAIP2) expression was down-regulated.

Infection up-regulated genes were (USP54, RHOT2 Mitochondrial Rho GTPase) while, (ATP6V1A) expression decreased. This data supports the previously reported notion that PIF induced protection against cell death is due to abnormal protein formation.
